# Identification of SARS-CoV-2-specific T cell and its receptor

**DOI:** 10.1186/s13045-024-01537-6

**Published:** 2024-03-27

**Authors:** Qian Zhang, Qing Liang, Rui Zhang, Nan Wang, Xu Xiao, Jiahao Shao, Kejia Wang

**Affiliations:** 1 Clinical Research Institute, The First Affiliated Hospital of Xiamen University, State Key Laboratory of Vaccines for Infectious Diseases, Xiang An Biomedicine Laboratory, School of Medicine, Xiamen University, Xiamen, 361102 Fujian China; 2 Department of Emergency Medicine, Xiang’an Hospital of Xiamen University, School of Medicine, Xiamen University, Xiamen, 361102 Fujian China; 3 National Institute for Data Science in Health and Medicine, Xiamen University, Xiamen, 361102 Fujian China; 4 Shanghai Engineering Research Center of Intelligent Education and Bigdata, Shanghai Normal University, Shanghai, 200234 China

**Keywords:** SARS-CoV-2, T cell immunity, T cell receptor, Immune repertoire

## Abstract

The T-cell receptor (TCR) repertoires exhibits distinct signatures associated with COVID-19 severity. However, the precise identification of vaccine-induced SARS-CoV-2-specific TCRs and T-cell immunity mechanisms are unknown. We developed a machine-learning model that can differentiate COVID-19 patients from healthy individuals based on TCR sequence features with an accuracy of 95.7%. Additionally, we identified SARS-CoV-2-specific T cells and TCR in HLA-A*02 vaccinated individuals by peptide stimulation. The SARS-CoV-2-specific T cells exhibited higher cytotoxicity and prolonged survival when targeting spike-pulsed cells in vitro or in vivo. The top-performing TCR was further tested for its affinity and cytotoxic effect against SARS-CoV-2-associated epitopes. Furthermore, single-cell RNA sequencing (scRNA-seq), immune repertoire sequencing (IR-seq) and flow cytometry were used to access vaccine-induced cellular immunity, which demonstrated that robust T cell responses (T cell activation, tissue-resident memory T cell (Trm) generation, and TCR clonal expansion) could be induced by intranasal vaccination. In summary, we identified the SARS-CoV-2-associated TCR repertoires profile, specific TCRs and T cell responses. This study provides a theoretical basis for developing effective immunization strategies.

**Supplementary Information:**

The online version contains supplementary material available at 10.1186/s13045-024-01537-6.

## To the editor

Coronavirus Disease 2019 (COVID-19) is a global public health concern caused by the severe acute respiratory syndrome coronavirus 2 (SARS-CoV-2). Despite significant emphasis on vaccine inoculation globally, vaccine-induced neutralizing antibody immunity alone has proven insufficient to prevent SARS-CoV-2 infection [1, 2]. Accumulating evidences demonstrate the critical role of coronavirus-specific T lymphocytes for recovery and long-term protection [3]. SARS-CoV-2 vaccination has triggered a robust and enduring T cell response that can effectively recognize variants from Alpha to Omicron [4]. Recent study indicates a disease severity-dependent TCR clonal expansion pattern in COVID-19 patients, demonstrating that the disease-specific TCRs is required for symptomatic relief [5]. However, the landscape of T-cell receptor (TCR) repertoires in COVID-19 and the TCRs responsible for recognizing SARS-CoV-2 remain uncertain [6, 7].

**Fig. 1:**
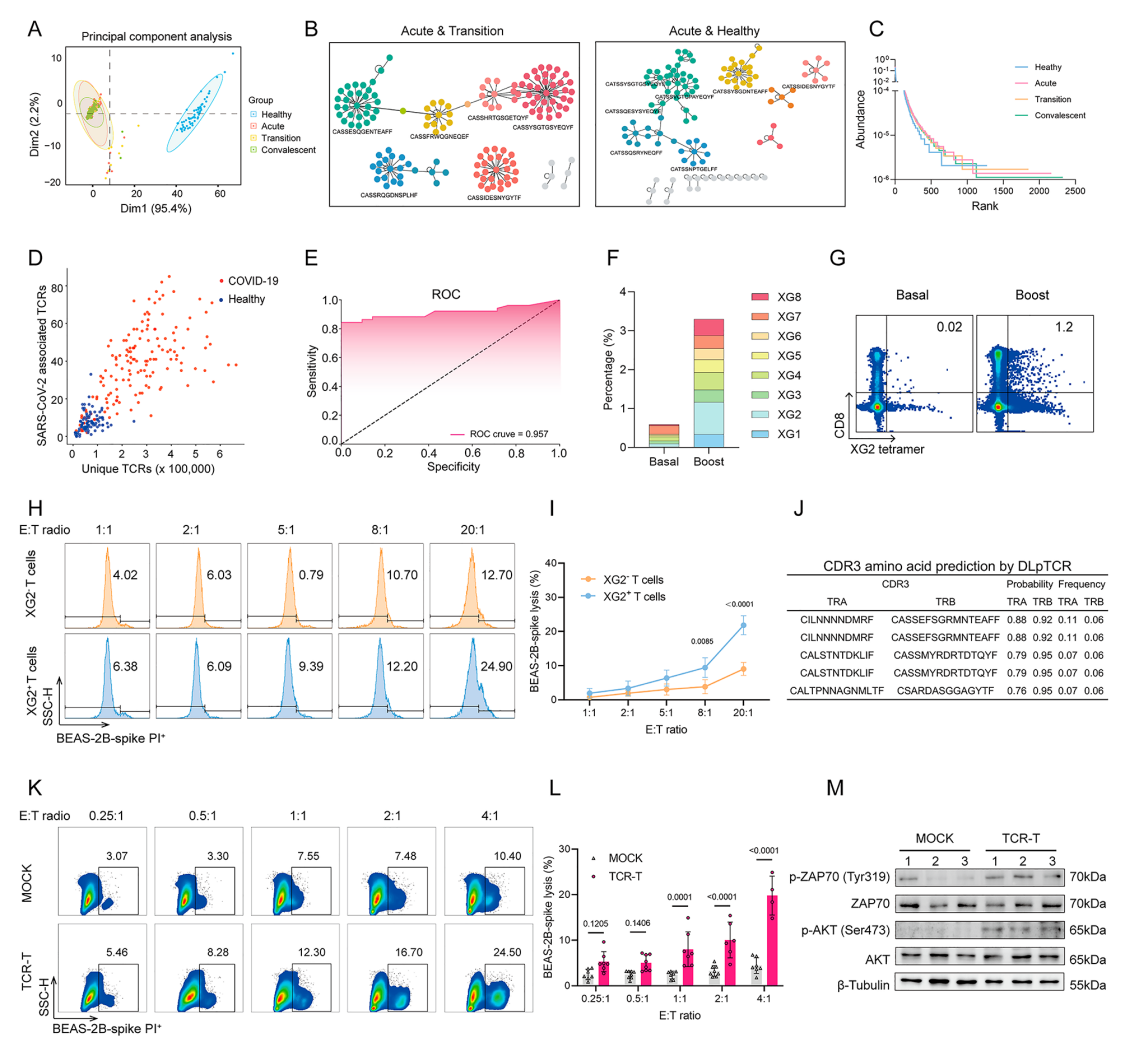
The characteristics of SARS-CoV-2-specific T cell and TCR repertoire. **A** Principal Component Analysis (PCA) visualization of TCR sequences obtained from the ImmuneACCESS and ImmuneCODE databases, comparing healthy donors (*n* = 54) with patients at different stages of infection **(**acute *n* = 103, transition *n* = 90, and convalescent *n* = 108**). B** Levenshtein distances depict TCR clone similarities between acute and transition groups and diversity in TCR clones between acute and healthy groups. **C** Rank-abundance curve illustrating TCR diversity. **D** Machine learning framework for analyzing TCR sequences from COVID-19 patients and healthy individuals. **E** Performance evaluation of machine learning models for predicting SARS-CoV-2 infection. **F** Bar graphs representing the proportion of peptide-specific CD8^+^ T cells in HLA-A*02^+^ healthy donors before and after immunization. **G** Representative flow plot showing the percentage of specific T cells (CD8^+^XG2^+^) after peptide stimulation. **H** Cytotoxic activities of XG2^+^ T cells against BEAS-2B-spike cells assessed at different effector/target (E/T) ratios. **I** Quantification of lysis rates when co-cultured with BEAS-2B-spike cells (*n* = 4). **J** Top 5 CDR3 amino acid sequences predicted by IR-seq and DLpTCR. **K** Cytotoxic activities of TCR-T cells against BEAS-2B-spike cells assessed by flow cytometry. **L** Quantification of TCR-T cell lysis rates when co-cultured with BEAS-2B-spike cells (*n* = 8). **M** Comparison of phospho-ZAP70, ZAP70, phospho-AKT, AKT, and β-Tubulin expression in TCR-T cells by immunoblot analysis (*n* = 3). Data are representative of at least three independent experiments

This study, as illustrated in Additional information, Fig. S1, was designed to address these uncertainties. Initially, we conducted a comprehensive analysis of peripheral blood TCR repertoire in various groups, including healthy controls and individuals at different stages of SARS-CoV-2 infection (comprising 54 healthy, 103 acute, 90 transition, and 108 convalescent patients), utilizing data from the ImmuneACCESS and ImmuneCODE databases (Additional information, Table S1). Comparison of TCR repertoire differences, the overlap ratio and complementary-determining region 3 (CDR3) amino acid usages between acute and transition groups (0.110) was more similar than others (Additional information, Fig. S2A, B). Besides, infected patients revealed significant differences in TCR repertoire distribution compared with healthy controls (Fig. 1A, B). Notably, TCR patterns in patients indicated a predilection for high-frequency clusters, while controls exhibited different TCR usage profiles characterized by a predilection for low-frequency clusters, attributed to increased TCRs diversity following SARS-CoV-2 infection (Fig. 1C; Additional information, Fig. S2C-E). Moreover, we developed a machine-learning model that could accurately differentiate COVID-19 patients from healthy individuals based on TCR sequence features, achieving an impressive area under the receiver operating characteristics (ROC) curve value of 95.7% (Fig. 1D, E). Intriguingly, we observed similarities in TCR repertoires when comparing TCR sequences after SARS-CoV-2 infection and vaccination, suggesting the potential for specific T cell and TCR identification post-SARS-CoV-2 vaccination (Additional information, Fig. S2F).

To identify SARS-CoV-2-specific T cells and TCRs, we employed a multiplexed peptide-MHC tetramer staining approach to screen 8 spike or nucleocapsid protein (XG1-XG8) for recognition by T cell responses with HLA allele HLA-A*02, the most common HLA class I allele in China [8]. Booster vaccinations notably enhanced T cell activation (Fig. 1F), with the SLSSTASAL peptide (XG2 peptide, one of peptide from spike protein) demonstrating the most robust expansion of CD8^+^XG2^+^ T cells and heightened cytokine expression (*IL2*, *GZMB*, *GZMK*, *IFNG* and *TNF*) (Fig. 1G; Additional information, Fig. S3A). To assess the cytotoxicity of XG2^+^ T cells, we co-cultured them with epithelial cells (BEAS-2B or SV-HUC-1) expressing spike protein by lentivirus (pCDH-EF1a-spike-GFP) infection. (Additional information, Fig. S3B). Compared to XG2^−^ T cells, XG2^+^ T cells exhibited higher cytotoxicity and prolonged survival when targeting spike-pulsed cells (Fig. 1H, I; Additional information, Fig. S3C). Immune repertoire sequencing (IR-seq) and a deep learning framework for predicting immunogenic peptide recognized by TCR (DLpTCR) approaches were used to determine specific TCR clonotype from XG2^+^ T cells (Additional information, Table S2). Compared with XG2^−^ T cells, XG2^+^ T cells showed the significant decrease in VJ and CDR3 amino acid usage after vaccination (Additional information, Figs. S3D-G). We identified the top 5 high-probability CDR3 amino acid sequences binding to the SLSSTASAL peptide (Fig. 1J). Subsequently, one of these high-probability TCRs (TRA CDR3, CILNNNNDMRF; TRB CDR3, CASSEFSGRMNTEAFF) was overexpressed in CD8^+^ T cells (Additional information, Fig. S3H, I), leading to enhanced cytolytic activity against target cells (Fig. 1K, L) with the elevated phospho-ZAP70 (Tyr319) and phospho-AKT (Ser473) (downstream of TCR signaling) (Fig. 1M).

**Fig. 2:**
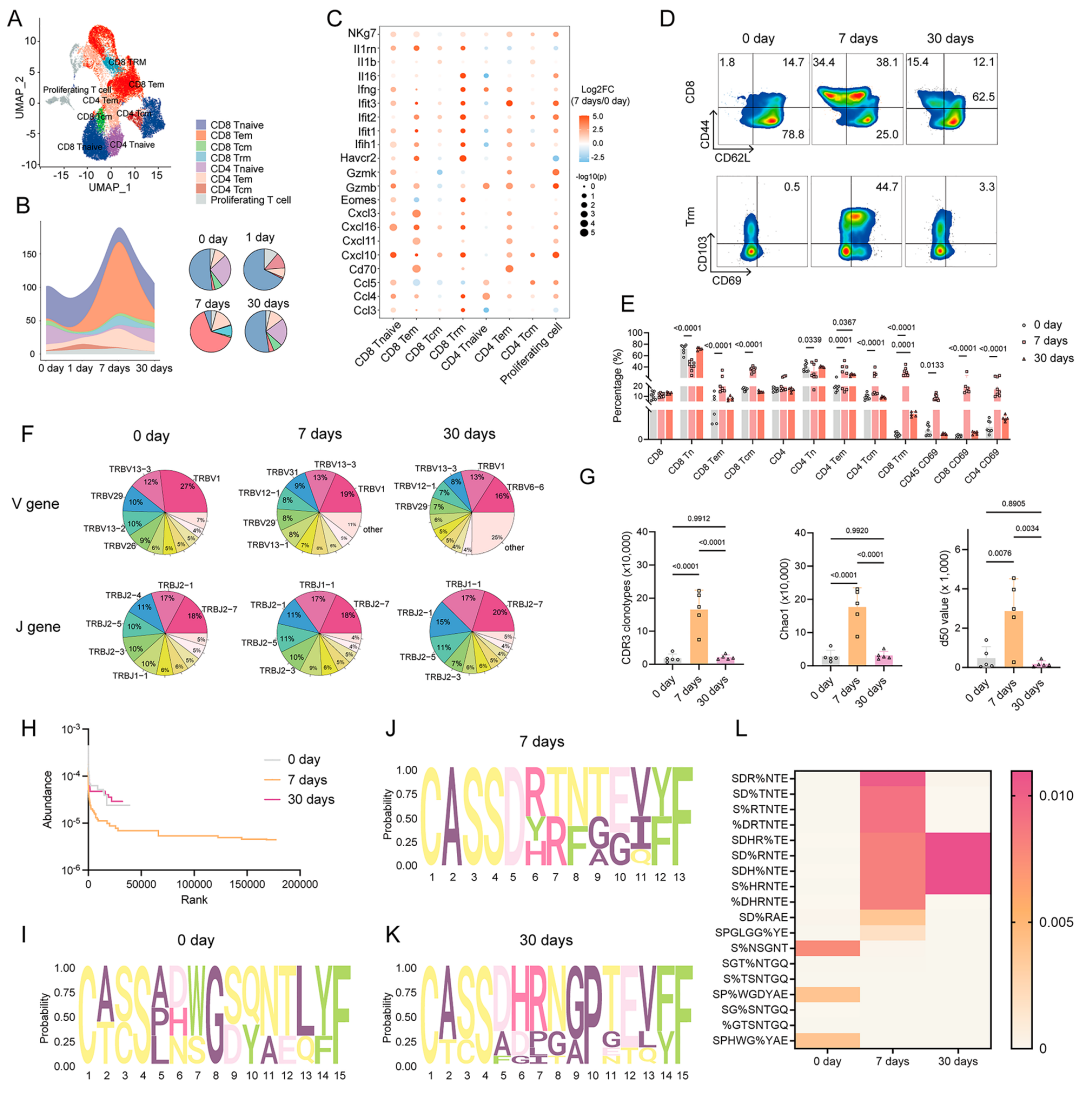
Intranasal immunization enhances T cell response. **A** UMAP plot of scRNA-seq displaying pulmonary T cell subsets at 0, 1, 7, and 30 days post intranasal immunization in mice (*n* = 3). **B** Proportion of T cell subsets in pulmonary tissue at indicated time-point. **C** Bubble plot showing the expression of T cell activation genes at 0 and 7 days post**-**immunization. **D** Representative flow plots depicting CD8^+^ memory T cells and Trm in pulmonary tissues. **E** The percentage of various T cell subsets in pulmonary tissue after vaccination (*n* = 8). **F** Analysis of TCR repertoires in pulmonary tissues by IR-seq (*n* = 5). Pie graph displaying the top10 V and J gene usages after 0 (left), 7 (middle), and 30 days (right) immunization. **G** TCR diversity based on CDR3 amino acid clonotypes (left), Chao1 (middle), d50 (right). **H** Rank abundance analysis of TCR clonotypes. **I-K** Bias analysis of CDR3 amino acid motif. **L** Heatmap hierarchical clustering of CDR3 amino acid sequences. Data are representative of three independent experiments. Data are representative of at least three independent experiments

To evaluate the T cell responses in the lower respiratory tract elicited by specific peptides, we immunized mice intranasally with the SLSSTASAL peptide (Additional information, Fig. S4A). Lung mononuclear cells were collected at 1, 7 and 30 days post-immunization for scRNA-seq and IR-seq (Additional information, Table S3, S4). Compared to non-immunized individuals, peptide-stimulated pulmonary tissues displayed increased fractions of total, central memory (Tcm), effector memory (Tem), and tissue-resident memory T cells (Trm) in the early days (1 and 7 days) (Fig. 2A, B; Additional information, Fig. S4B) without inducing tissue injury or inflammatory responses (Additional information, Fig. S4C-E). These T cells also exhibited high activation genes and various cytokine genes expressions (*Ccl5, Cxcl10, Cxcl16, Gzmb, Gzmk, Ifng, and Nkg7*) after 7 days post-immunization, similar to XG2^+^ T cells from humans (Fig. 2C; Additional information, Fig. S4F, G). Flow cytometry further confirmed a significant increase in the percentage of memory T cells and T cell activation (Fig. 2D, E; Additional information, Fig. S4H). Although the effect of T cell activation diminished after 30 days post-immunization, Trm cells were still detectable (Fig. 2D, E; Additional information, Fig. S4I-K). Additionally, we evaluated the pulmonary TCR repertoire on 0 day, 7 days, 30 days after intranasal immunization. Vaccination enhanced TRBV12-1 usage and reduced TRBV1 usage (Fig. 2F). Similar with TCRs expansion in COVID-19 patients, antigenic stimulation significantly augmented TCRs diversity on 7 day post-immunization (Fig. 2G, H), leading to similar CDR3 amino acids usage (including SHDR%TE, SD%RNTE, SDH%NTE, and S%HRNTE) (Fig. 2I-L). Taken together, antigen exposure induced significant expansion of TCR clonotypes in local pulmonary tissues, suggesting that epitope-specific Trm responses could provide long-term protection against SARS-CoV-2 infection.

In summary, our study introduces a machine-learning approach capable of accurately predicting COVID-19 infection severity based on TCR sequence features. We successfully identified SARS-CoV-2-specific T cells and their CDR3 sequences from human peripheral blood and observed a robust memory T cell response in local pulmonary tissues. Furthermore, we cloned specific TCR sequences in CD8^+^ T cells and established highly efficient TCR-T cells. Our research introduces an autonomous TCR screening platform capable of identifying precise TCR sequences that bind to specific HLA-peptide complexes. Leveraging this platform, we can similarly pinpoint neoantigen-associated TCRs in various diseases, including cancer, infections, and autoimmune conditions.

### Electronic supplementary material

Below is the link to the electronic supplementary material.

Supplementary Material 1

Supplementary Material 2

Supplementary Material 3

Supplementary Material 4

Supplementary Material 5

Supplementary Material 6

Supplementary Material 7

Supplementary Material 8

Supplementary Material 9

Supplementary Material 10

## Data Availability

No datasets were generated or analysed during the current study.

## Abbreviations

COVID-19: Coronavirus Disease 2019
SARS-CoV-2: Severe acute respiratory syndrome coronavirus 2
TCR: T cell receptor
CDR3: Complementary-determining region 3
ROC: Receiver operating characteristics
PCA: Principal component analyses
IR-seq: Immune repertoire sequencing
DLpTCR: Deep learning framework for predicting immunogenic peptide recognized by TCR
scRNA-seq: Single-cell RNA sequencing
Tcm: Central memory T cell
Tem: Effector memory T cell
Trm: Tissue-resident memory T cells
