# Supplementary material for: Identification of SARS-CoV-2-specific T cell and its receptor
Source: J Hematol Oncol. 2024 Mar 27;17:15. doi: 10.1186/s13045-024-01537-6 (PMC10976674; doi:10.1186/s13045-024-01537-6)
Supplement: Supplementary file 1 — Supplementary Material 1 [file 13045_2024_1537_MOESM1_ESM.docx]

**Additional Information**

**Materials and Methods**

**Learning network model**

TCR repertoires datasets of all Coronavirus Disease 2019 (COVID-19) patients were sourced from ImmuneACCESS (https://clients.adaptivebiotech.com/ immuneaccess) under the category "SARS-CoV-2 and symptom", encompassing 103 acute, 90 transition, and 108 convalescent patients. TCR repertoires datasets from 54 healthy donors and 199 vaccinated individuals (28 days post-vaccination) were obtained from ImmuneCODE (https://www.adaptivebiotech.com/immunecode). To construct a machine learning model for identifying individual exposure to SARS-CoV-2, we adopted a previously described approach. Briefly, the model was trained for classification tasks, distinguishing between healthy donors and COVID-19 patients. We utilized a total of 253 TCR clones from COVID-19 patients as the positive training set and another 16,408,195 TCR clones from healthy donors as the negative training set. Initially, we identified shared TCR repertoires occurring in more than ten individuals. Subsequently, we conducted Fisher's precision probability tests on a 2×2 contingency table to identify a set of TCR clones that exhibited a significantly higher incidence among phenotype-positive individuals. TCR clones meeting the criterion of *P*<0.05 were considered SARS-CoV-2-associated TCR clones. To validate the learning framework, we employed another dataset from ImmuneCODE as a testing cohort. The classification accuracy between the two groups was assessed using Receiver Operating Characteristics (ROC) curves.

**Human samples**

The APC-peptide-tetramer was created by adding APC-Streptavidin (Cat: 405207, Biolegend) to the p-MHC monomer at a molar ratio 1:0.87. This process was performed in six fractions, each incubated for ten minutes. Peripheral blood mononuclear cells (PBMCs) were isolated from HLA-A2^+^ volunteers' peripheral blood using Ficoll (Cat: P8900, Solarbio) following the provided protocol. These cells were then cultured in X-VIVO15 medium (Cat: 04-418Q, LONZA) supplemented with 50 ng/ml OKT3 (Cat: 317301, Biolegend), 1 μg CD28 (Cat: 302934, Biolegend), 10 ng/ml IL-2 (Cat: CX66, Novoprotein), 10 ng/ml IL-7 (Cat: CX47, Novoprotein), and 10 ng/ml IL-15 (Cat: C016, Novoprotein). Subsequently, PBMCs from the individuals were initially stained with HLA-DR (Cat: 307618, L243, 1:50, Biolegend), CD38 (Cat: 303530, HIT2, 1:100, Biolegend), and CD8 (Cat: 300908, HIT8a, 1:100, Biolegend) for 20 minutes at 4°C in the dark. The cells were washed twice with 1 mL PBS containing 0.2% fetal bovine serum (FBS) (Cat: A0500-3011-2871, Cegrogen) and stained with XG2 tetramer for 1 hour at 4°C in the dark. After staining, the cells were resuspended in 300 μl of buffer and acquired using the Beckman CytoFlex S (BECKMAN COULTER, United States). Informed consent was obtained from all individuals, and the procedures were approved by the Ethics Committee of Xiamen University in China.

**Peptide-specific T cells culture and isolation**

Peptide-specific T cells were stimulated for 7-10 days by adding one of the following peptides at a concentration of 10 ng/ml: XG1 (RLNEVAKNL), XG2 (SLSSTASAL), XG3 (SIIAYTMSL), XG4 (FIAGLIAIV), XG5 (RLQSLQTYV), XG6 (RLDKVEAEV), XG7 (LLLDRLNQL), or XG8 (YLQPRTFLL), to the T cell culture medium. These peptides were synthesized by SciLight Biotechnology, LLC and corresponded to the ancestral Wuhan sequence's SARS-CoV-2 spike or nucleocapsid protein. After stimulation, T cells were stained with peptide-tetramer for 1 hour at 4°C in the dark. Tetramer-positive or negative T cells were sorted using the Beckman MoFlo Astrios EQS (BECKMAN COULTER, United States).

**Cell lines**

The SV-HUC-1 cell line was provided by Dr. Z.Y. Shi (Xiamen University) and cultured in Ham's F-12K medium (Cat: YC-3034, Basalmedia) supplemented with 10% FBS and 1% Penicillin/Streptomycin (P/S) (Cat: c3420-0100, Vivacell). The 293T cell line was obtained from Dr. J. Lin (Xiamen University). The BEAS-2B cell line was purchased from Procell Life Science & Technology Co., Ltd. The BEAS-2B-spike cell line was generated in the lab by transduction with pCDH-CMV-spike-EF1a-GFP lentiviral vectors (LVs). All BEAS-2B and SV-HUC-1 cell lines mentioned above and 293T cells were cultured in Dulbecco's Modified Eagle Medium (DMEM) (Cat: 01-052-IACS, BI) supplemented with 10% FBS and 1% P/S. All procedures involving LVs were conducted in a Biosafety Level 2 (BSL2) laboratory.

**Mice model**

The T cell response analysis of PBMCs and pulmonary lymphocytes was triggered by intranasal immunization. C57BL/6 mice (6-8 weeks old) were immunized with a two-dose regimen intranasal vaccine (Beijing Wantai Biological Pharmacy Enterprise Co., Ltd., Beijing, China) at 0 and 14 days, with each dose consisting of 50 μl (1×10^6^ PFU/ml). The control group received 50 μl of PBS at the same time. All experimental mice were housed in pathogen-free animal rooms at the Animal Care Centre of Xiamen University. At 7 and 30 days post-immunization, mice were sacrificed to collect blood and pulmonary tissue for further T cell flow cytometry analysis, single-cell RNA sequencing (scRNA-seq), and immune repertoire sequencing (IR-seq). All mouse experiments in this study were approved by the Committee on Animal Care.

**Tissue dissociation and flow cytometry**

Mice pulmonary tissue samples were cut into pieces and washed twice with Fluorescence Activating Cell Sorter (FACS) buffer. The tissues were then digested in 4 ml PBS supplemented with 1 mg/ml Dispase II (neutral protease, grade II, Cat: 49442078001, Roche), 300 μg/ml DNase I (Cat: 11284932001, Roche), and incubated at 37°C for 2 hours with gentle shaking. The tissue was further processed with the gentleMACS dissociator (Miltenyi Biotech, Germany). After digestion, the tissues were washed once and treated with ACK (Cat: R1010, Solarbio) at 4°C for 2 minutes to lyse the red cells. Then, single cell suspensions were obtained for T-cell staining. All single cells from the mice's pulmonary tissue were resuspended in 100 μl FACS buffer and stained with LIVE/DEAD Fixable Aqua Dead Cell Stain Kit (Cat: L23102, Invitrogen). For the surface stain, cells were washed twice with FACS buffer, resuspended in 100 μl PBS, and stained with CD45.2 (Cat: 109827, Clone: 104, 1:100, Biolegend), CD4 (Cat: 100421, Clone: GK1.5, 1:100, Biolegend), CD8 (Cat: 100711, Clone: 53-6.7, 1:100, Biolegend), CD44 (Cat: 103047, Clone: IM7, 1:200, Biolegend), CD62L (Cat: 104427, Clone: MEL-14, 1:100, Biolegend), CD69 (Cat: 104527, Clone: H1.2F3, 1:50, Biolegend), and CD103 (Cat: 121405, Clone: 2E7, 1:100, Biolegend) antibodies at 4°C for 30 minutes in the dark. After staining, cells were washed twice with FACS buffer and resuspended in 300 μl FACS buffer. All samples were acquired on Beckmann CytoFlex LX (BECKMAN COULTER, United States).

**Enzyme-linked immunosorbent assay (ELISA)**

ELISA was used to detect the secretion of TGF-β, IL-10, MMP19, IL-1α, IL-1β, and TNF-α in epithelial cells (CD326^+^) and endothelial cells (CD31^+^) 24 hours after stimulation with 1 MOI intranasal vaccine. Samples were pipetted into coated ELISA plates and incubated with reagents according to the manufacturer's instructions (AIMENG YOUNING, Shanghai, China). The final signal was revealed by adding a colored reaction product (TMB). Colorimetric reactions were stopped by adding an equal volume of 1M H_2_SO_4_ solution, and the plates were read at an optical density of 450 nm. Cytokine concentrations were calculated based on the standard curve.

**TCR IR-seq**

The process of IR-seq was performed as previously described. Briefly, total RNA from sorted T cells was extracted using the SPARKeasy RNA extraction kit (Cat: AC0205-B, SPARKJade) following the protocol. Then, 200 ng of total RNA was reverse-transcribed into cDNA using Hifair III 1st Strand cDNA Synthesis SuperMix for qPCR Kit (Cat: 11141ES60, Yeasen) on a C1000 TouchTM Thermal Cycler (Bio-Rad Inc., Hercules, CA, United States). Two-round nested amplicon arm-PCR with specific primers was performed using 2 × Taq master Mix (Cat: K1082, Thermo Fisher) as previously described. The 100-250 bp amplicons were extracted from 2.0% agarose gels and purified using the AxyPrep DNA Gel Extraction Kit (Hlingene, Shanghai, China). Purified amplicons were paired-end sequenced (PE250) on the Illumina platform according to standard protocols. The sequencing data were saved in FASTQ format, and subsequent analysis exclusively employed the high-quality filtered data, excluding any sequences of poor quality. All TCR repertoires mentioned in this study were extracted from clean raw data using MiXCR (version 3.1.5). The R package immunarch (version 0.9.0) was used to analyze the immune repertoire, including V(D)J usage, complementary-determining region 3 (CDR3) amino acid diversity, clonotype distribution, and tracking on the R (version 4.1.2) platform.

**scRNA-seq and data processing**

The single cell suspension of mice's pulmonary tissue was prepared as described in the "Tissue Dissociation and Flow Cytometry" section. scRNA-seq libraries were prepared according to instructions using the ChromiumTM Single cell 5′ Reagent Kit for the Chromium platform (10×Genomics, California, United States). Then, the scRNA-seq libraries were sequenced on the Illumina HiSeq X Ten platform, and Cell Ranger (v6.1.1) was used to prepare count matrices for the gene expression library.

**Real-time quantitative PCR (qPCR)**

To further assess the specific T cell function, total RNA was extracted from XG2^+^ T cells and XG2 tetramer negative T cells using the SPARKeasy Total RNA extraction kit (AC0402, SparkJade). Then, 50 ng of total RNA was reverse-transcribed into cDNA using the Hifair III 1st Strand cDNA Synthesis SuperMix for qPCR Kit (Cat: 11141ES60, Yeasen) on a C1000 TouchTM Thermal Cycler (Bio-Rad Inc., Hercules, CA, United States). Reverse transcription-polymerase chain reaction was performed using Hieff qPCR SYBR Green Master Mix (No Rox) (Cat: 11201ES08, Yeasen) with forward and reverse primers and the CFX96 Real-Time PCR Detection System (Bio-Rad Inc., Hercules, CA, United States). All primers used are listed in Additional information, Table S5.

**XG2-specific T cell cytotoxic assay**

BEAS-2B cells were co-cultured with XG2^+^ T cells or XG2^-^ T cells at 37°C for 5 hours at different effector-to-target (E:T) ratios after staining with CFSE (Cat: S19285, Shanghai Yuanye Bio-Technology) for 10 minutes at 37°C. BEAS-2B-spike and SV-HUC-1-spike cells were directly co-cultured with the T cells using the same processing method. After co-culture, 25 μg/ml propidium iodide (Cat: R20287, Shanghai Yuanye Bio-Technology) was used to quantify the ratio of target cell death through flow cytometry analysis. All samples were acquired on Beckman CytoFlex S (BECKMAN COULTER, United States).

**Immunoblotting**

XG2^+^ T cells and XG2^-^ T cells were lysed in RIPA buffer (Solarbio) containing protease and phosphatase inhibitors (Bimake). The proteins were separated by sodium dodecyl sulfate-polyacrylamide gel electrophoresis (SDS-PAGE) and transferred to PVDF membranes (Millipore). The membranes were then incubated with primary antibodies against ZAP70 (Cat: R26132, 1:1000, Zenbio), phospho-ZAP70 (Cat: 310211, 1:1000, Zenbio), AKT (Cat: ET1609-51, 1:1000, HUABIO), and phospho-AKT (Cat: 110852-R0069, 1:600, SinoBiological) overnight at 4°C. After incubation with the corresponding secondary antibodies, the protein bands were visualized by adding the HRP membrane substrate (Cat: E1060, LABLEAD) and then scanned using Azure 300.

**Generation of TCR-T cells**

For the detection of priority CDR3 amino acid sequences, XG2^+^ T cells were sorted for IR-seq, followed by a deep learning framework for predicting immunogenic peptide recognized by TCR (DLpTCR, http://jianglab.org.cn/DLpTCR/). We searched for amino acid sequences of the V and J genes on IMGT (https://www.imgt.org/IMGTrepertoire/) and replaced the CDR3 amino acid sequences (α chain: FGAGTRLTVKP; β chain: FGQGTRLTVV), obtaining α and β sequences. α and β sequences were linked by the P2A sequence (GSGATNFSLLKQAGDVEENPGP) and then synthesized and subcloned into the pCDH lentiviral vector. The lentivirus (LV) was generated in 293T cells. In short, 293T cells were plated in a 10 cm dish to achieve 90% confluence for transfection. On the day of transfection, 5 μg of Lentivector, 3.6 μg of pLP1, 3.6 μg of pLP2, and 1.8 μg of pLP-VSVG plasmid were added to 1 mL of Opti-MEM medium (Cat: 31985062, Invitrogen), and 42 μg of PEI (Cat: P4000, LABLEAD) was added to another 1 mL of Opti-MEM medium. After 10 minutes, the PEI-medium mixture was dropped and vortexed into the plasmid mix medium to create the PEI-DNA complex. The complex was incubated for 15 minutes at room temperature and then added to the 293T culture for 48 hours. All supernatants were collected and centrifuged for 90 minutes (20,000 g at 4℃) to obtain lentiviral particles. The LV was resuspended at 4℃ with X-VIVO 15 medium and stored at -80℃. When PBMCs were isolated, T cells were cultured at a density of 1 × 10^6^ cells per well and then added to 100 μl of TCR LV for 72 hours to generate the TCR-T cells.

**Quantification and statistical analysis**

Flow cytometry data were analyzed in FlowJo vX software (Becton, Dickinson and Company, United States). Data plots were created in GraphPad Prism 9 (GraphPad Software, La Jolla, CA). Paired comparison data of T cell plotted in the linear chart were analyzed using Wilcoxon tests and expressed as the geometric mean ± standard deviation (SD). Group comparisons were calculated using Two-way ANOVA analysis and expressed as mean ± SD.

**Supplementary figure legends**

**
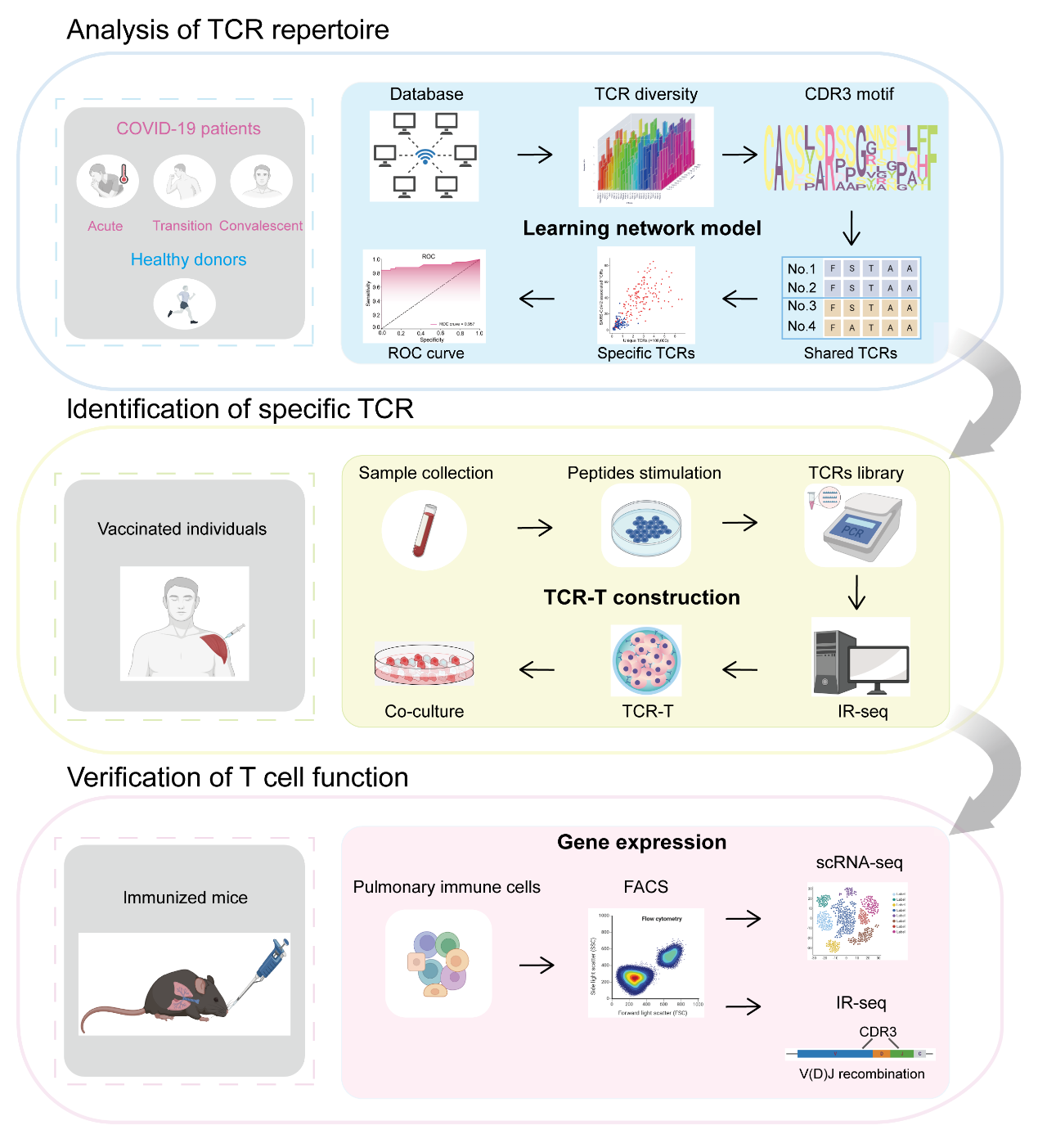
Fig. S1** The experimental design in this study.

**
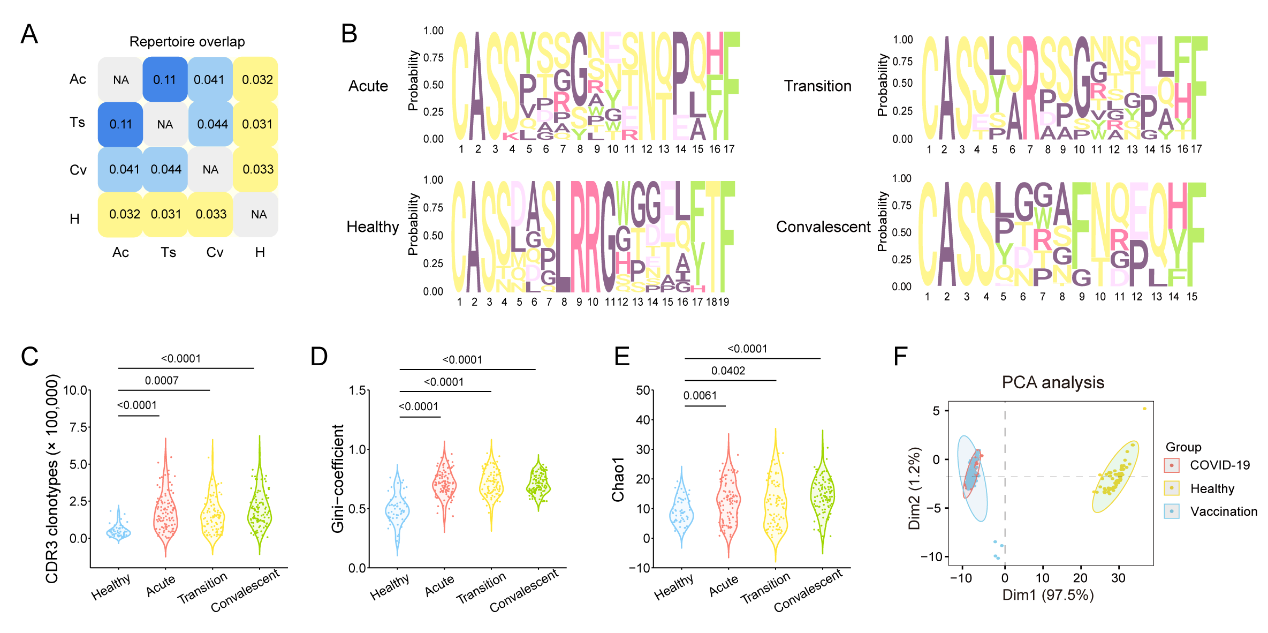
Fig. S2** Analysis of TCR repertoires from public databases. **A** Overlaps of TCR clones among healthy individuals, acute, transition, and convalescent patients (n=54, 103, 90, 108). **B** CDR3 amino acid motif bias. **C-E** Comparison of TCR diversity based on clonotypes **(C)**, Gini-coefficient **(D)**, and Chao1 **(E)**. **F** PCA visualization of TCR repertoire profiles among COVID-19 patients, healthy donors, and vaccinated individuals (n=301, 54, 199).

**
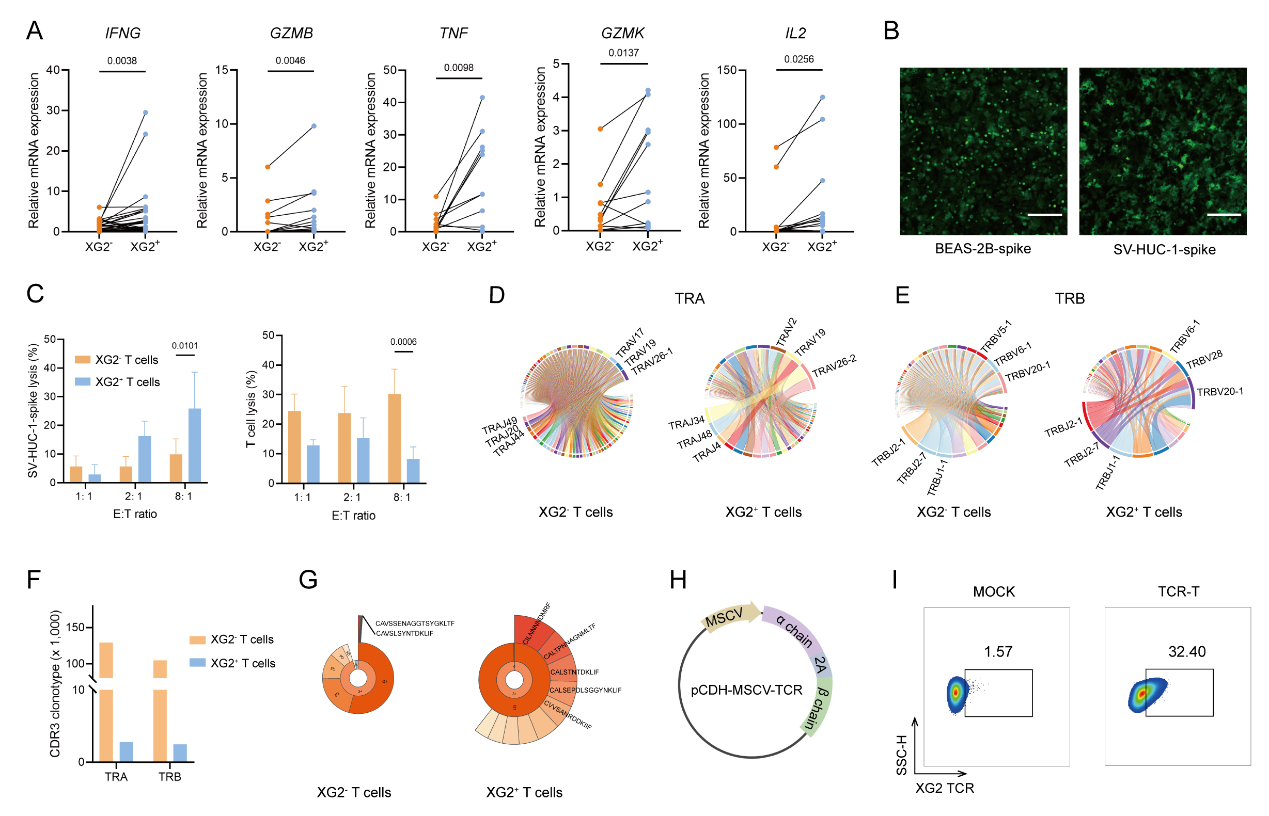
Fig. S3** Characterization of spike-specific T cells. **A** qPCR analysis of relative mRNA expression of cytokines in XG2^-^ and XG2^+^ T cells. **B** Representative fluorescent images showing successful spike expression in BEAS-2B and SV-HUC-1 cells. **C** Cytolytic activities of XG2^+^ T cells against SV-HUC-1-spike cells at different E/T ratios (n=5). **D, E** Circus plot displaying VJ pair segments of TRA **(D)** and TRB **(E)** in XG2^-^ and XG2^+^ T cells. **F** Clonotypes of CDR3 amino acids in XG2^-^ and XG2^+^ T cells. **G** Doughnut plot depicting CDR3 amino acid count, frequency, and diversity. The innermost layer represents the clonotypes with low-frequency. The second layer has 5 parts from Q1–Q5, which represent the top 20% (Q1), 20%–40% (Q2), 40%–60% (Q3), 60%–80% (Q4), and 80%–100% (Q5) clonotypes to the "3+" set, and the outermost layer indicates the top 5 clonotypes of Q1. **H** Representation of pCDH-MSCV-TCR lentiviral vector. **I** Representative images of XG2-specific TCR expression in T cells. Data are representative of four to five independent experiments.


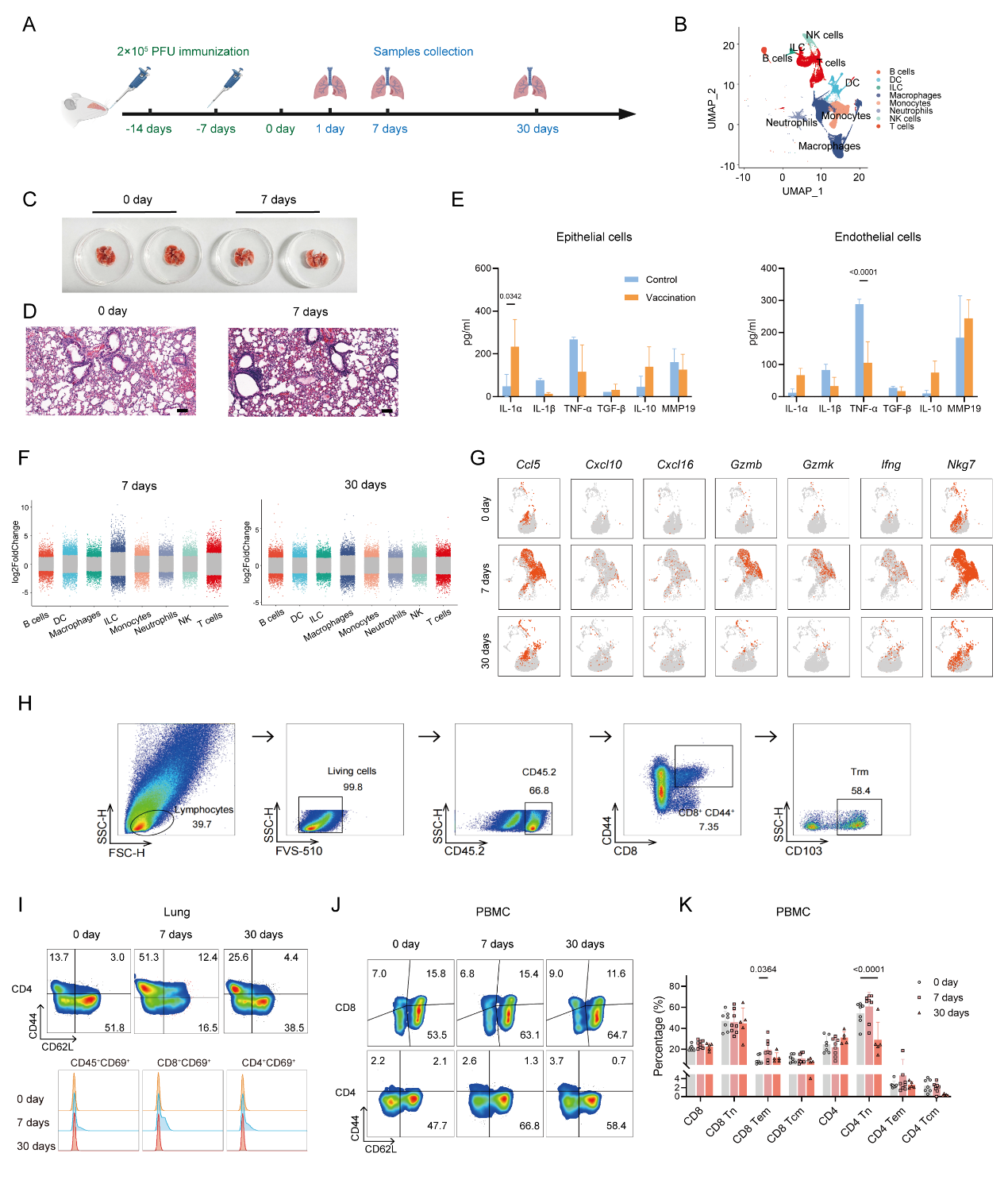


**Fig. S4** scRNA-seq analysis of pulmonary immune cells after intranasal immunization in mice. **A** Flowchart of intranasal immunization. **B** UMAP plot displaying immune cells in mouse pulmonary tissue. **C** Gross visualization of pulmonary tissue after intranasal immunization (n=3). **D** Representative HE staining images of pulmonary tissue (scale bar, 100 μm**).** **E** Secretion of TGF-β, IL-10, MMP19, IL-1α, IL-1β**,** and TNF-α by epithelial cells (CD326^+^) and endothelial cells (CD31^+^) analyzed by ELISA (n=4). **F** Differential gene analysis among immune cell types post 7 and 30 days immunization. **G** UMAP visualization of selected marker gene expression projection. **H** The gating strategy of Trm. **I, J** Representative plots of memory (CD44^+^CD62L^+^) and activated (CD69^+^) T cells in pulmonary tissues (**I**) and PBMCs (**J**). **K** Percentage of T cell subsets in PBMCs post 0, 7**,** and 30 days immunization (n=8). Data are representative of three to five independent experiments.

Table S1 The characteristic of SARS-CoV-2 associated TCRs.

Table S2 High-probability CDR3 sequence of XG2^+^ T cells.

Table S3 scRNA-seq analysis of pulmonary immune cell.

Table S4 IR-seq analysis of pulmonary TCR repertoires.

Table S5 qPCR primers in this study.
