# Supplementary figures and images for: Identification of SARS-CoV-2-specific T cell and its receptor
Source: J Hematol Oncol. 2024 Mar 27;17:15. doi: 10.1186/s13045-024-01537-6 (PMC10976674; doi:10.1186/s13045-024-01537-6)

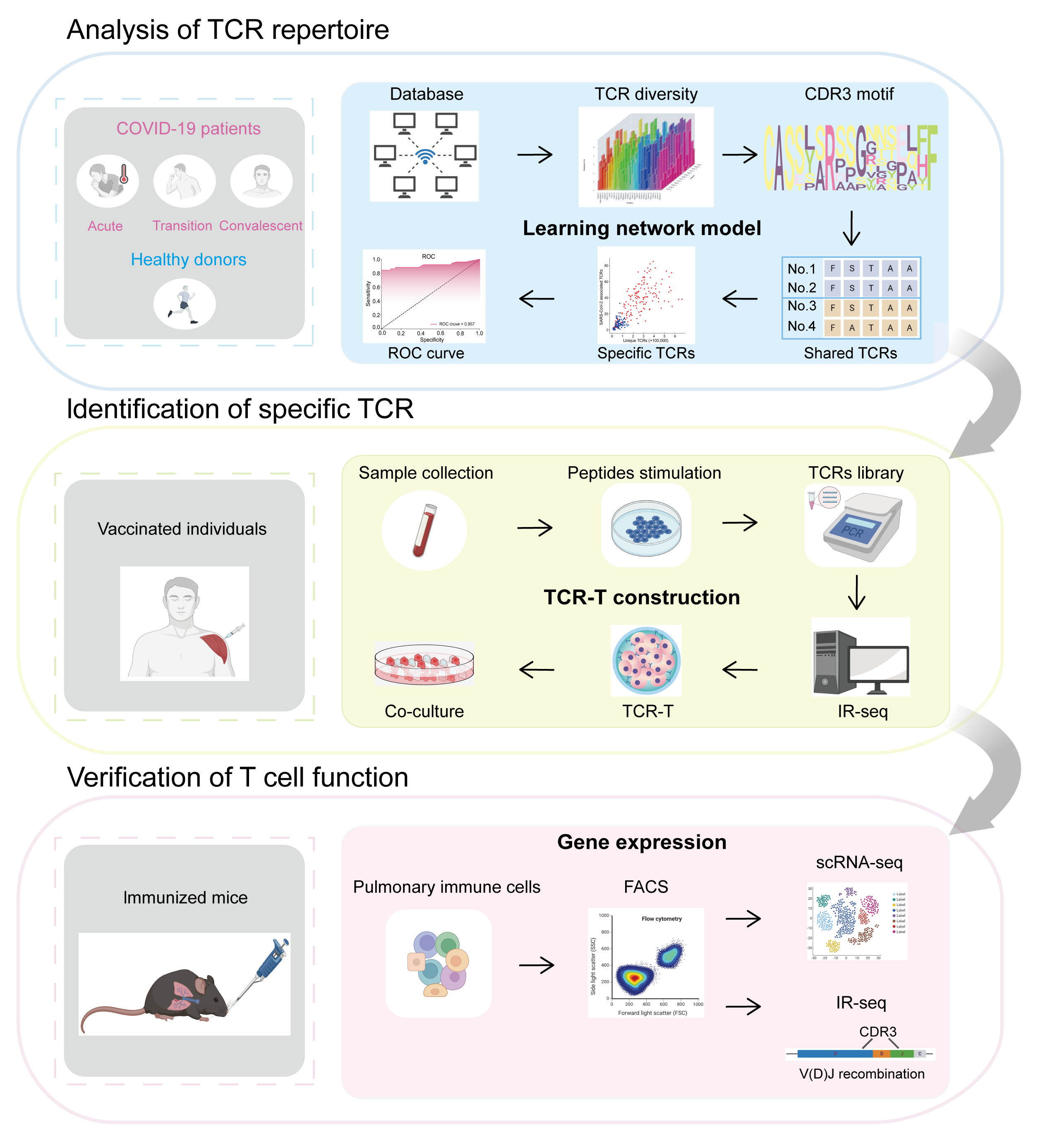

Supplement: Supplementary file 2 — Supplementary Material 2 [file 13045_2024_1537_MOESM2_ESM.tif]

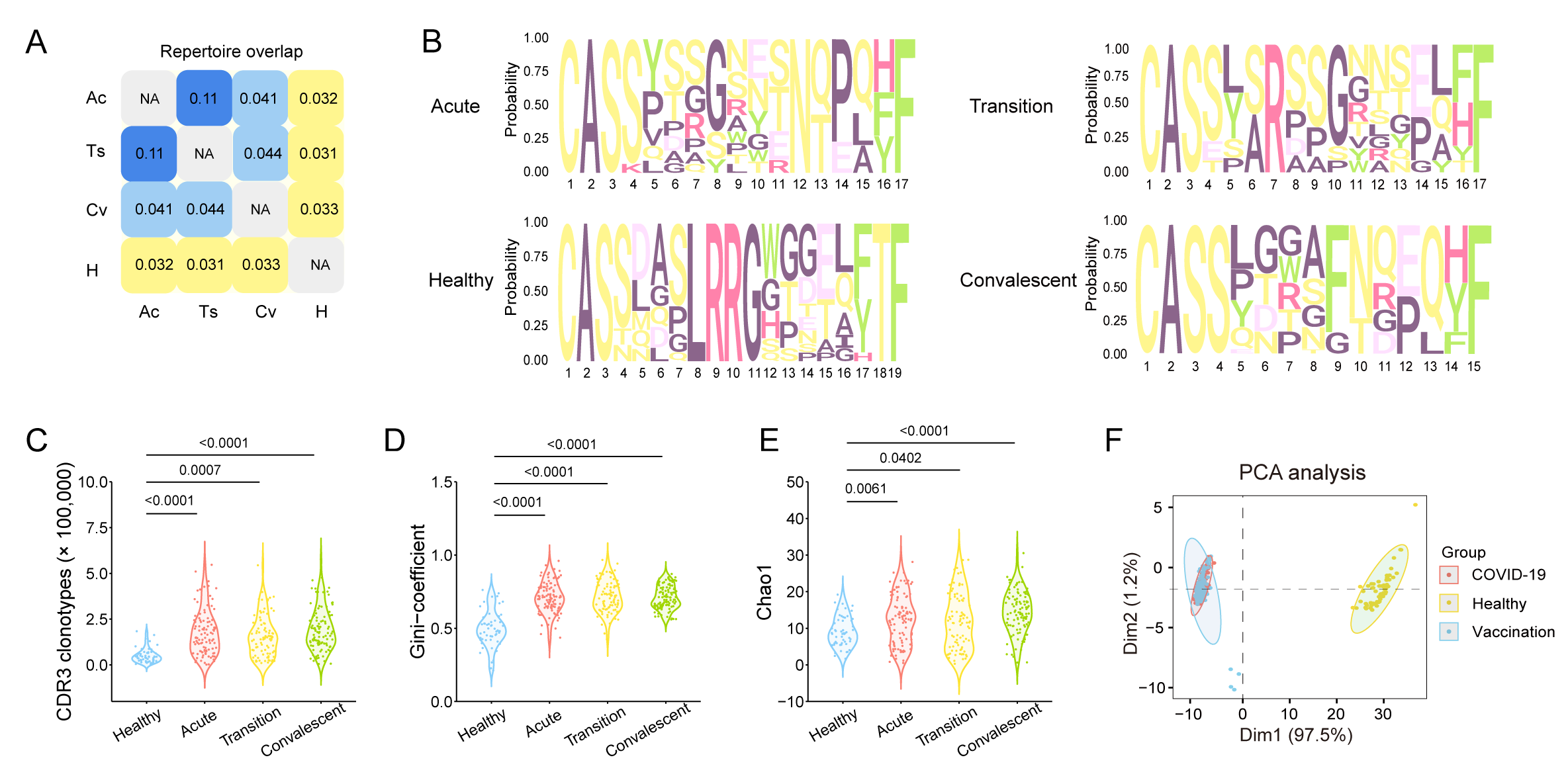

Supplement: Supplementary file 3 — Supplementary Material 3 [file 13045_2024_1537_MOESM3_ESM.tif]

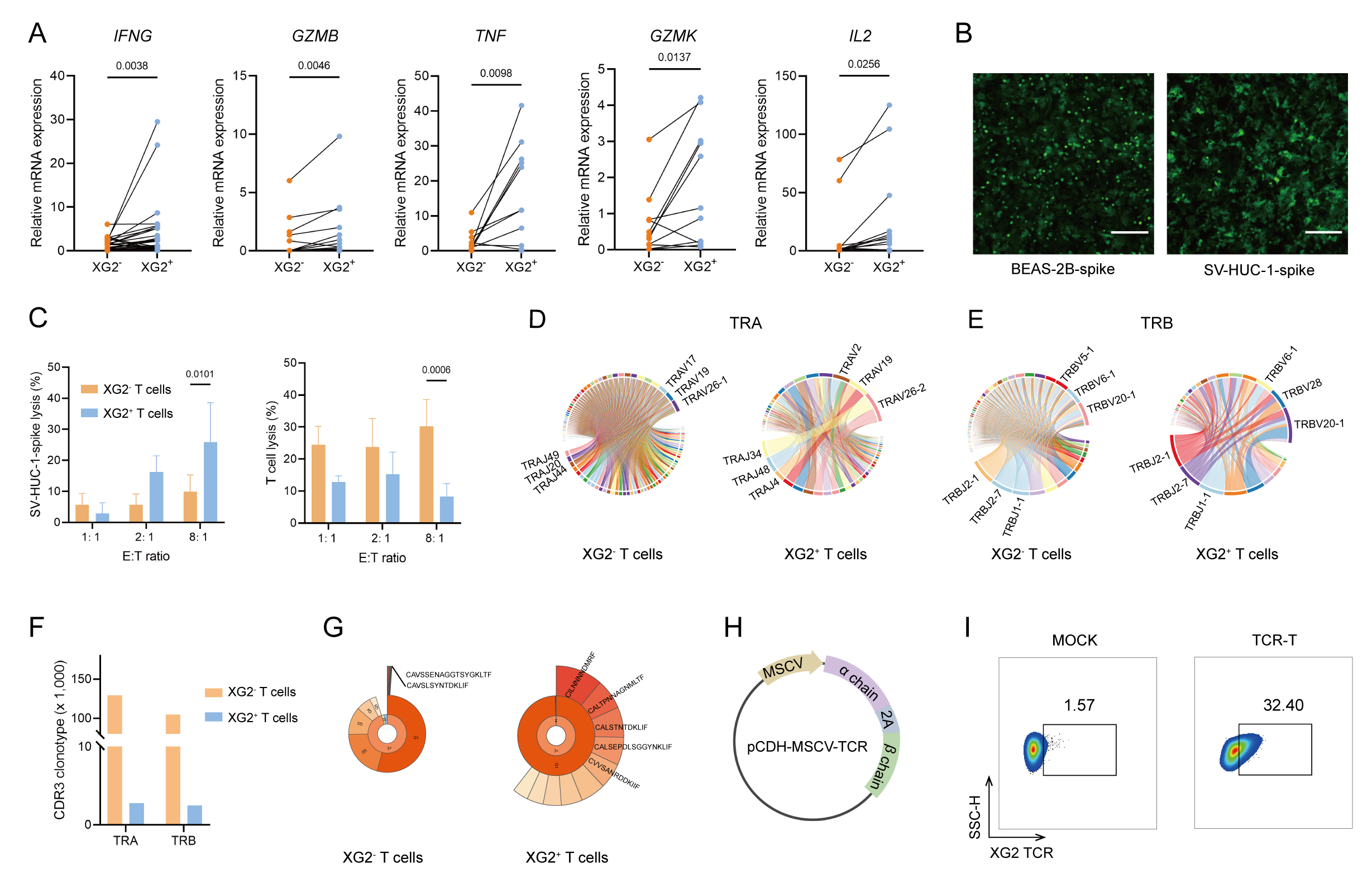

Supplement: Supplementary file 4 — Supplementary Material 4 [file 13045_2024_1537_MOESM4_ESM.tif]

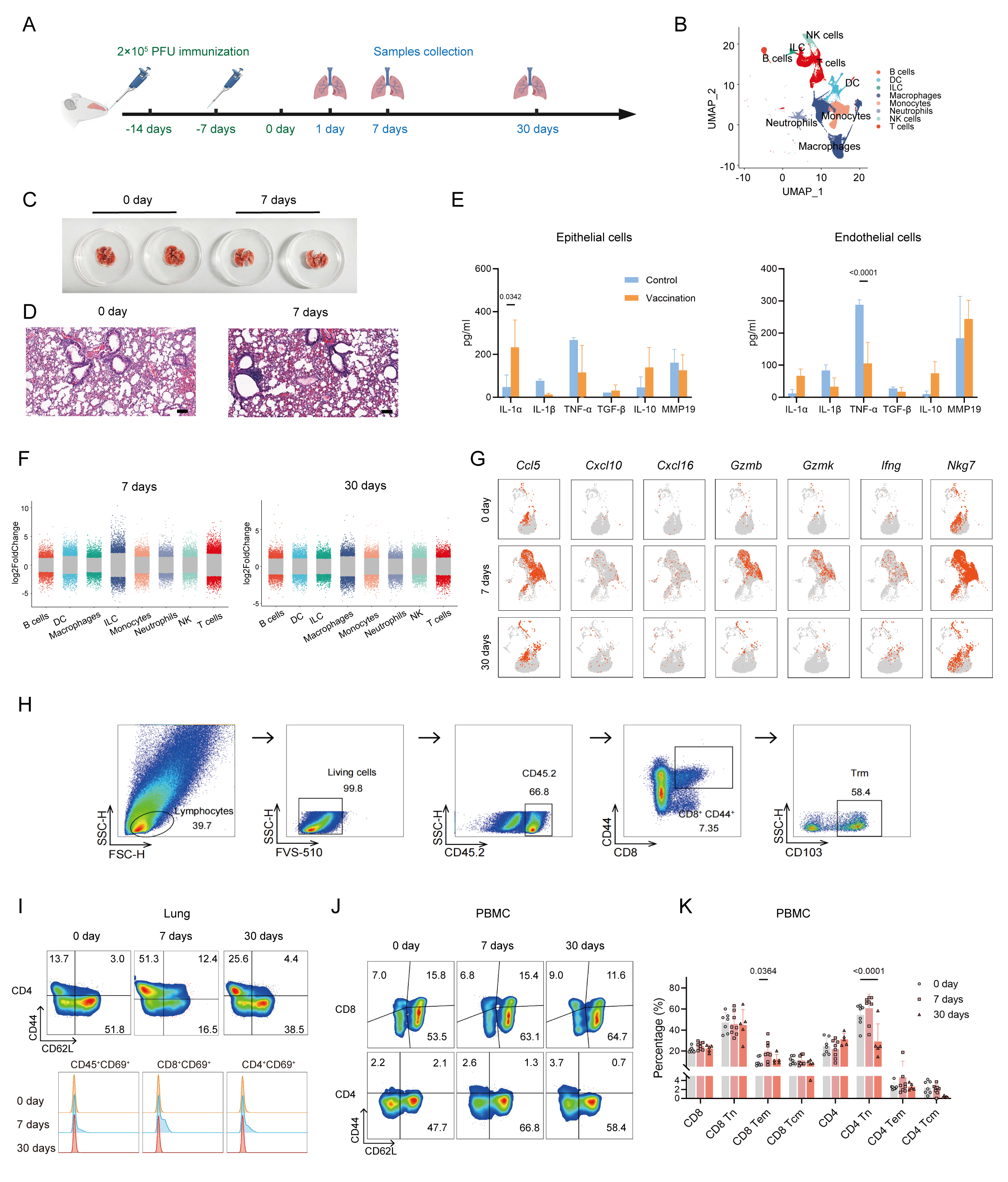

Supplement: Supplementary file 6 — Supplementary Material 6 [file 13045_2024_1537_MOESM6_ESM.tif]
